# *Demodex folliculorum* Infestation in Meibomian Gland Dysfunction Related Dry Eye Patients

**DOI:** 10.3389/fmed.2022.833778

**Published:** 2022-02-24

**Authors:** Yiran Hao, Xiaoyu Zhang, Jiayu Bao, Lei Tian, Ying Jie

**Affiliations:** ^1^Beijing Tongren Eye Center, Beijing Tongren Hospital, Beijing Institute of Ophthalmology, Capital Medical University, Beijing, China; ^2^Beijing Ophthalmology and Visual Sciences Key Laboratory, Beijing Tongren Hospital, Capital Medical University, Beijing, China; ^3^Beijing Advanced Innovation Center for Big Data-Based Precision Medicine, Beihang University & Capital Medical University, Beijing, China

**Keywords:** dry eye, *Demodex folliculorum* infestation, meibomian gland dysfunction, lid margin abnormality score, meibum expression, meibomian gland dropout, chalazion

## Abstract

**Objective:**

To report the influence of *Demodex folliculorum (D. folliculorum)* infestation in patients with meibomian gland dysfunction (MGD) related dry eye and the associations of the infestation with MGD related dry eye.

**Methods:**

Eyelashes (three from the upper eyelid and three from the lower eyelid) from 119 eyes of 119 patients diagnosed with MGD related dry eye were examined under a light microscope. There were 68 eyes of 68 patients with MGD related dry eye and *D. folliculorum* infestation (*Demodex* positive group) and 51 eyes of 51 patients without infestation (*Demodex* negative group). All patients completed an Ocular Surface Disease Index (OSDI) questionnaire and underwent tests for dry eye and MGD. The tests included fluorescein tear breakup time (TBUT), corneal fluorescein staining, Schirmer I test (SIT), lid margin abnormalities, meibum expression assessment, and meibomian gland dropout.

**Results:**

The scores for OSDI, corneal fluorescein staining, lid margin abnormalities, meibum expression, and meibomian gland dropout were significantly higher (all *P* < 0.05), while TBUT was significantly shorter in the *Demodex* positive group compared to the *Demodex* negative group (*P* = 0.020). The SIT values did not significantly differ between groups. Chalazion was significantly more prevalent in the *Demodex* positive group. The number of *D. folliculorum* was positively correlated with all three MGD parameters (*P* ≤ 0.035), OSDI; corneal fluorescein scores, and it was inversely correlated with BUT. The correlation for SIT was *R*^2^ = 0.075 (*P* = 0.064).

**Conclusion:**

*Demodex folliculorum* infestation is possibly one of the key contributors in the pathogenesis of MGD related dry eye, and a higher prevalence of chalazion was found in *D. folliculorum* infected patients. The possible causal role of *D. folliculorum* infestation needs to be further studied.

## Introduction

*Demodex* can have different effects on the ocular surface, including anterior blepharitis, posterior blepharitis accompanied by meibomian gland dysfunction (MGD), ocular rosacea, keratitis, and so on (1). There are two types of *Demodex* being identified in humans: *Demodex folliculorum* and *Demodex brevis. Demodex folliculorum (D. folliculorum)* is generally found around the root of the eyelashes as well as the lash follicles, while *Demodex brevis (D. brevis)* primarily infest the deeper sebaceous glands (2, 3). Previous studies have reported that *Demodex* infestation is related to ocular diseases including blepharitis, dry eye, conjunctivitis, corneal injury, and so on (4, 5). To be specific, *Demodex folliculorum* is more likely to cause anterior blepharitis with eyelash involvement while *Demodex brevis* may lead to posterior blepharitis more often, and the streptococci and staphylococci reside on the surface of the mites will lead to blepharitis both anteriorly and posteriorly (2, 3, 6). However, the role of *Demodex* infestation in the pathogenesis of chronic blepharitis is still controversial (1, 3, 6).

Dry eye disease (DED) is considered a type of multifactorial disease accompanied by various ocular symptoms. The pathogenesis of which mainly includes the imbalance of the microenvironment of the ocular surface and the instability of the tear film. According to the TFO DEWS II (2017), the condition could also be along with inflammation and damage of the ocular surface, as well as neurosensory disorders (7).

Meibomian glands, the largest sebaceous glands in the body, produce the main component of the tear film to maintain a stabilized ocular surface (8). They synthesize and secrete mixed lipids known as meibum, which are delivered through orifices located anterior to the mucocutaneous junction (9, 10). Diseases of the meibomian gland can be subdivided into focal lesions (hordeolum or chalazion) and diffuse lesions (MGD). MGD is a chronic disease of the meibomian glands, whose pathological basis includes the obstruction of the terminal duct of the glands with or without changes in the amount or quality of the meibum (11). When MGD happens, multiple factors including enhanced tear evaporation, hyperosmolarity, increased pro-inflammatory mediators in tears, and reduced lubrication between the eyelids and the eyeball will break the stability of tear film and disequilibrate the balance of the ocular surface, thus contributing to a series of ocular symptoms and signs, which overlap with DED (12). Therefore, MGD is considered to be a key factor of evaporative dry eye.

The reported prevalence of MGD was found to vary considerably. To be specific, the prevalence of which in the Shihpai Eye Study in Taiwan is 60.8%, in the Beijing Eye Study is 68.3%, and in the Singapore Malay Eye Study is 56.3% (13–15). MGD is more prevalent in the Asian populations and has been associated with pinguecula, anterior blepharitis, contact lens use, and infestation with *Demodex* mites (4, 13). Liang et al. reported a high prevalence of demodicosis, especially *Demodex brevis*, in patients with chalazion (4). However, the frequency of *Demodex* infestation in patients with dry eye accompanied by MGD and the association between *Demodex* infestation and Meibomian function have not been studied in detail.

As the detection rate of *D. brevis* is quite low in meibomian glands and could be affected by various factors, the objective of this study was to evaluate the function of meibomian glands and the ocular surface characteristics in MGD related dry eye patients with or without *Demodex folliculorum* infestation, to investigate the influence of anterior blepharitis on the ocular signs and symptoms of MGD related dry eye patients.

## Methods

All patients diagnosed with dry eye in the cornea clinic of the Tongren Eye Hospital, Beijing, between August 2020 and May 2021 were eligible for inclusion. Patients who are willing to participate in the study, with the same ethnicity (Chinese) and over 18 years old, were included after signing the informed consent. Subjects who previously underwent corneal or ocular surgery, had any ocular diseases other than DED, MGD, or *Demodex* infestation, had any other systemic, dermatologic, or rheumatologic diseases known to impact the tear film, had worn contact lens in the past 24 h, or underwent any treatment related to *Demodex* within 2 weeks, on medication recently were excluded from the study. A total of 119 eyes of 119 patients diagnosed with dry eye disease (DED) associated with meibomian gland dysfunction (MGD) who met the inclusion and exclusion criteria were included. The institutional review board of the Beijing Tongren Hospital, Beijing, China approved the study as TRECKY2021-065 in 2021, and all participants signed informed consent. The study was conducted in accordance with the Declaration of Helsinki.

The DED diagnosis was made according to the TFO DEWS II diagnostic methodology report (2017), which implies: (1) Ocular Surface Disease Index (OSDI) questionnaire ≥13 scores; (2) fluorescein tear film breakup time (TBUT) ≤ 5 s; (3) a non-anesthesia Schirmer I test value ≤ 5 mm/5 min; (4) being reported with corneal fluorescein staining (16).

The diagnosis of MGD was confirmed based on the international workshop on meibomian gland dysfunction: report of the diagnosis subcommittee (2011), which includes the following: (1) clinical signs: meibomian gland dropout, altered meibomian gland secretion, and changes in lid morphology; (2) symptoms of global discomfort including redness and swelling, itching, irritation, soreness, and so on (11).

All patients completed the OSDI survey and underwent the following ocular surface evaluation: (1) tear function assessment: fluorescein tear breakup time (TBUT), Schirmer I test (SIT); (2) assessment of meibomian gland function: lid margin examination, meibum quality, meibomian gland dropout, examination (or recorded diagnosis) of chalazion; (3) assessment of *Demodex* infestation as detailed below.

### Ocular Surface Evaluation

#### Ocular Surface Disease Index

All patients were asked to complete the OSDI survey before clinical examinations, providing a score ranging from 0 (no symptoms) to 100 (severe symptoms).

#### Corneal Fluorescein Staining

Corneal fluorescein staining was determined after recording TBUT (described below) and was graded from 0 to 15 according to the National Eye Institute/Industry grading scale (NEI). Each section of the cornea was graded as 0 (no staining), 1 (mild, with <10 scattered staining dots), 2 (moderate, between 10 and 30 dots), and 3 (severe, with over 30 dots, confluent staining, or presence of corneal filaments), and the total of five sections was recorded (17).

### Tear Function Assessment

#### Fluorescein Tear Breakup Time (TBUT)

Fluorescein dye was used to evaluate corneal staining and TBUT. A moistened aseptic fluorescein strip was dipped in the inferior fornix. The time interval between the last blink and the appearance of the first random dry spot on the corneal surface, being observed through a slit-lamp microscope with a cobalt-blue filter, was recorded by a stopwatch (16). The average of three consecutive TBUT tests was recorded. Corneal fluorescein staining was assessed after TBUT measurements and was graded according to the NEI scale.

#### Schirmer I Test (SIT)

A standard 5 × 40 mm Schirmer test strip was applied over the middle and outer third of the inferior lid. Patients were required to keep their eyes closed for 5 min, during which time the amount of wetting was recorded (18).

### Assessment of Meibomian Gland Function

Meibomian gland assessment was carried out as previously described in the literature (19).

#### Lid Margin

The following lid margin abnormalities were recorded and scored from 0 to 4: irregularity, vascular engorgement, obstruction of meibomian gland orifices, anterior or posterior displacement of the mucocutaneous junction (20).

#### Meibum Expression

The quality of the meibum was semi-quantitatively graded in eight glands of the central third of the lower eyelids. To be specific, Grade 0: clear; Grade 1: cloudy; Grade 2: cloudy with particulate material and Grade 3: inspissated and toothpaste-like (11).

#### Meibomian Gland Dropout

All subjects underwent infrared imaging of the meibomian glands in both upper and lower eyelids with the Keratograph 5M (K5M; Oculus Optikgeräte GmbH, Wetzlar, Germany). After everting the upper and lower eyelids, the areas of partial or complete loss of the meibomian glands were scored according to criteria proposed by Arita et al. (20). The sum of the meiboscores for both upper and lower eyelids was recorded for each eye.

### Demodex Examination

**Figure 1:**
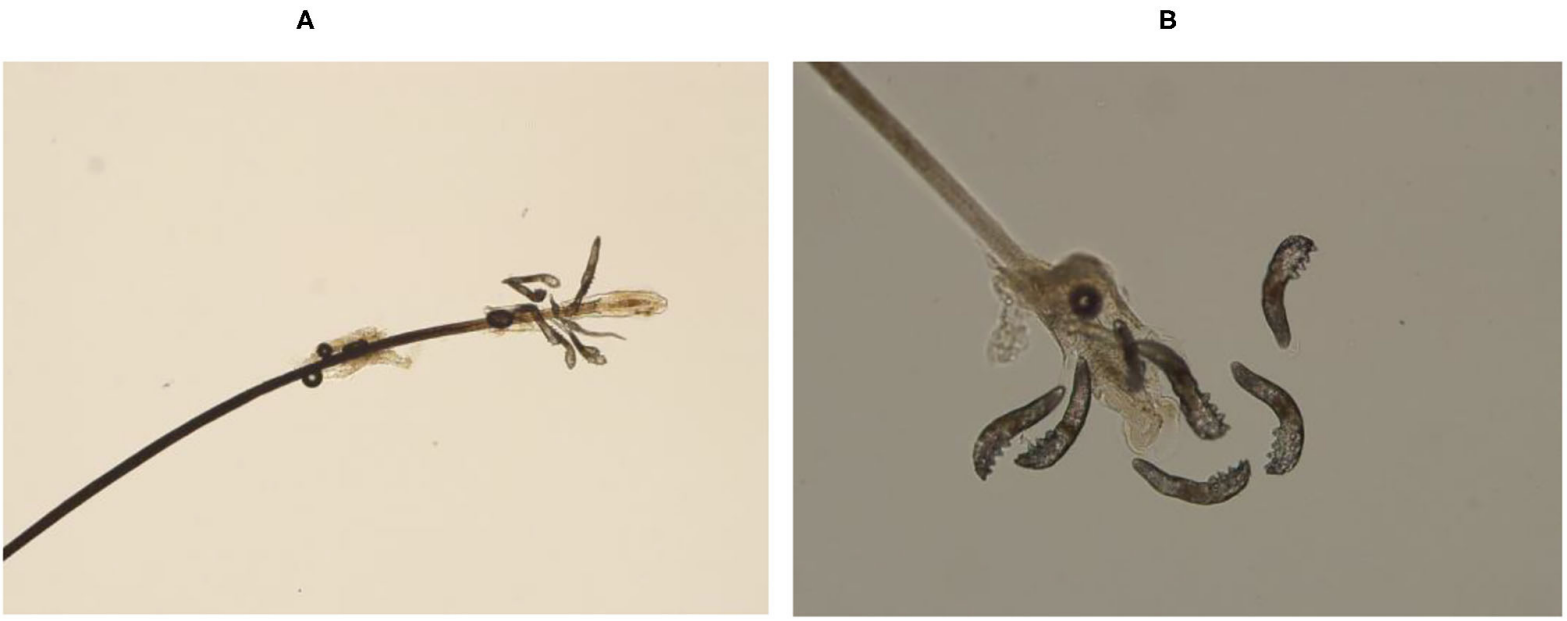
*D. folliculorum* on the root of the eyelash. *Demodex* inside dandruff can be observed clearly both at low (×100) and high (×400) magnification.

A modified sampling and counting method reported in the previous study (4) was used. Six eyelashes (3 each from the upper and lower eyelids) from the right eye of each patient were removed from and examined for *Demodex* species under a light microscope. The three lashes from each lid were selected from the nasal, middle, and temporal side; lashes were likely to have a higher tendency to harbor *Demodex—*those with cylindrical dandruff-like material at the base or those with a different color/brittle appearance were selected (21). Before epilation, the lash was rotated to bring any *Demodex* closer to the surface. The epilated lashes were placed on a glass slide, a drop of cedar oil was gently added to the lash, and the slide was examined under the microscope without a coverslip. A technician blinded to the clinical findings performed the microscopic examination and mite count. Figure 1 shows images of *Demodex* seen on the root of a lash.

The patients were analyzed based on the presence or absence of *Demodex* on the eyelashes. The right eye from each patient was selected for analysis.

### Statistical Analysis

All analyses were performed using SPSS version 17.0 (SPSS, Inc., Chicago, IL, USA). Data were tested for normality by the Kolmogorov-Smirnov test, and the mean and standard deviation (SD) were displayed. Welch's modified Student's two-sample *t*-test and the Wilcoxon rank-sum test were used to evaluating the differences between groups. A Chi-square test was carried out to assess the difference in gender distribution. Linear regression analysis was performed to estimate the association between various factors. A *P* < 0.05 was considered statistically significant.

## Results

### Demographics

A total of 119 patients with MGD related dry eye who visited the Tongren Hospital were included in the study. After taking a series of ocular surface evaluations and *Demodex* examination described previously, subjects were subdivided into two groups according to the positivity of *Demodex* infestation. Sixty-eight eyes of 68 dry eye patients with MGD accompanied by *D. folliculorum* infestation were enrolled in the *Demodex* positive group, and 51 eyes of 51 dry eye patients with MGD related dry eye alone were enrolled in the *Demodex* negative group. The characteristics of the study population are shown in Table 1. The mean age of patients was 40.32 ± 12.88 years (range 19–65 years) with 22 men and 46 women in the *Demodex* positive group, while the mean age of patients was 39.65 ± 13.98 years (range 20–66 years) with 17 men and 34 women in the *Demodex* negative group. There was no difference between the mean age and gender composition between the two groups (age, *P* = 0.785; gender, *P* =0.910).

**Table 1:** Characteristics of the study population.

**Parameters**	***Demodex* positive group [range] (*n* = 68)**	***Demodex* negative group [range] (*n*= 51)**	***P*-value**
Age (year)	40.32 ± 12.88 [19–65]	39.65 ± 13.98 [20–66]	0.785
Gender (male/female)	22/46	17/34	0.910
OSDI (score)	45.12 ± 18.53 [14–90]	35.78 ± 18.24 [12–65]	0.007
TBUT (s)	4.18 ± 1.79 [2–9]	5.01 ± 2.04 [1.96–9.85]	0.020
SIT (mm/5 min)	8.15 ± 4.06 [1–19]	7.16 ± 5.21 [1–17]	0.246
Fluorescein score	4.52 ± 2.4 [0–10]	2.19 ± 1.28 [0–4]	<0.001
*Demodex* number of eyelids	7.53 ± 4.43 [3–21]	–	–
Chalazion, n (%)	35.29%	17.65%	0.033

*OSDI, the Ocular Surface Disease Index; TBUT, Fluorescein tear breakup time*.

All clinical indexes (OSDI, SIT, TBUT, and corneal staining) were significantly better in the *Demodex* negative group compared to the *Demodex* positive group. The prevalence of chalazion was significantly higher in patients with *Demodex* infection (*P* = 0.033).

### Meibomian Gland Indices

The prevalence of MGD was significantly higher in patients with *Demodex* infection (*P* = 0.002). In addition, lid margin abnormality score and meiboscore were significantly higher, and the expressed meibum was significantly worse in the *Demodex* negative group compared to the *Demodex* positive group (all *P* < 0.05, Table 2).

**Table 2:** Meibomian Gland Function Indices in *Demodex* positive and *Demodex* negative group.

**Parameters**	***Demodex* positive group [range] (*n* = 68)**	***Demodex* negative group [range] (*n* = 51)**	***P*-value**
Lid margin abnormality score	2.32 ± 0.72 [0–3]	1.53 ± 0.7 [0–3]	<0.001
Meibum score	2.06 ± 0.99 [0–3]	1.39 ± 1.02 [0–3]	<0.001
Meiboscore	3.54 ± 1.33 [2–6]	2.67 ± 1.74 [0–6]	0.0023

*MGD, Meibomian gland dysfunction*.

### Association Between Demodex Infestation, Ocular Surface, and Meibomian Gland Indexes

The number of *Demodex* was significantly associated with OSDI (*R*^2^ = 0.075, *P* = 0.024) and corneal fluorescein score (*R*^2^ = 0.144, *P* = 0.001) and inversely correlated with TBUT (*R*^2^ = 0.072, *P* = 0.027). The association with SIT was not statistically significant (*R*^2^ = 0.075, *P* = 0.064). A significant positive correlation was observed between the number of *Demodex* and all three MGD parameters (Lid margin abnormality score, *R*^2^ = 0.065, *P* = 0.035; meibum scores, *R*^2^ = 0.303, *P* < 0.001; Meiboscore, *R*^2^ = 0.232, *P* < 0.001).

## Discussion

It has been well-established that *Demodex* can infest eyelash follicles; however, its role in blepharitis remains controversial (22). Previous studies have shown that many infestations are asymptomatic, which could be related to the number of *Demodex* present (6, 23). *Demodex* may also worsen coexisting lid-margin diseases such as anterior blepharitis and posterior blepharitis, including MGD. A 60% prevalence of *Demodex* in lashes has been reported in patients with MGD vs. 18% in control subjects free of lid and margin disease (24). An over-reproduction of *Demodex* may cause lid-margin infestation, leading to irritation symptoms of ocular surface such as itching, a foreign-body sensation, or stinging (22). It has been recommended that *Demodex* infestation should be suspected in all patients with symptomatic advanced blepharitis, which is accompanied by abnormal fluorescein tear breakup time (22). As MGD is a key contributor to evaporative dry eye, a thorough examination of ocular symptoms, ocular signs, and meibomian gland function is necessary to evaluate the influence of *Demodex* infestation on MGD and dry eye to illuminate the role of *Demodex* in blepharitis.

Our study explored the relationship between the density of *Demodex* infestation with ocular symptoms and MGD function. We reported that the OSDI, corneal fluorescein score, the prevalence of chalazion and MGD, as well as the three MGD parameters measured, were significantly higher in the *Demodex* positive group, and the TBUT was significantly shorter in the *Demodex* positive group compared to the *Demodex* negative group. Our findings were consistent with the outcomes of previous studies (22, 25, 26), which indicated that ocular *Demodex* infection might be related to ocular discomfort and ocular surface damage. A previous study has clarified that *Demodex* can consume the lining of hair follicles and generate debris and waste accumulated at the root of the lashes, thus forming cylindrical dandruff (3). Another possible pathogenesis is that the mites mechanically obstruct the orifices of meibomian glands (1, 3). Both mechanisms lead to changes in the quality and quantity of glandular secretion, resulting in the alteration of the tear film, symptoms of eye irritation, clinically apparent inflammation, and ocular surface disease diagnosed as MGD or DED (27). Also, those abnormalities and dysfunction of the meibomian gland and ocular surface could be reflected by ocular parameters and meibomian gland indices that we have already studied. Apart from the causes of the mites themselves, the bacteria residing on the surface or inside themselves might be another possible pathogenesis. The staphylococci and streptococci on the surface of *Demodex* and the bacillus inside the *Demodex* as well as the toxins expressed by the organisms could activate the inflammatory cascade, contributing to the blepharitis both anteriorly and posteriorly and lead to the disorder of ocular surface and meibomian glands (1, 6, 28). Although previous studies reported that *D. folliculorum* and *D. brevis* were tend to parasite and cause blepharitis in different location, our results showed that the chronic blepharitis caused by *D. folliculorum* could also affect the function of ocular surface by the mechanisms discussed above and lead to a series of ocular signs and symptoms.

Our results also revealed that the number of *Demodex* was positively correlated with all MGD indices in the *Demodex* positive group. However, the minimum number required to produce symptoms is still unknown, and it is likely to be different in different patients.

In this study, we also tested the difference in the prevalence of chalazion with *Demodex* infestation. We found that the prevalence of chalazion was significantly higher in patients with *Demodex* infestation, which is consistent with the outcome from Liang et al. (4) who reported a high prevalence of demodicosis (69%) in those with chalazion compared to healthy controls (20.3%). They also reported an increased recurrence rate in those with infestation (33.3 vs. 10.3%) (4). Moreover, Yam et al. found a high prevalence of 72.9% of *Demodex* infestation in adult patients with recurrent chalazion (29). It has been shown that the chitinous exoskeleton of *Demodex* may act as a foreign body and produce granulomatous inflammation, which may implicate chalazion (4, 30). A recent study indicated that *Demodex* was also related to recurrence of chalazion after surgical excision (31). If *Demodex* is not radically eliminated, it cannot continue to clog the meibomian gland, resulting in abnormal meibum and leading to recurrent chalazion. Age is a key risk factor for MGD that has been proved to affect meibomian gland dropout, with the elderly showing a higher dropout rate (22, 32). To rule out the difference in age as a reason for the observed differences in the meibomian gland dropout between groups, we selected relatively young patients with a similar mean age between the two groups. While gender has also been associated with MGD dropout, there was a similar gender distribution between the two groups (33, 34).

The present study has several limitations. First, as it is an observational cross-sectional study, it cannot certainly assure whether *Demodex* infestation caused MGD and DED. Although the association between the number of mites and meibomian gland function indices and ocular surface parameters as well as biologic plausibility is suggestive of a causal role, it still merits further investigation. Also, we did not formally calculate the sample size. So, despite that our results are statistically significant, the sample could be considered relatively small. Apart from that, it is inevitable that false-negative results may appear during *Demodex* detection, for the six eyelashes removed from each patient is randomized and it is unavoidable to epilate uninfected lashes while infected lashes remained, which will cause false-negative result and affect the accuracy of the outcome.

In conclusion, our study indicated that all dry eye parameters and MGD indices were worse in the *Demodex* positive group than in the *Demodex* negative group. Specifically, the scores of OSDI, corneal fluorescein, lid margin abnormality, and meibum were significantly higher, and TBUT was significantly shorter in the *Demodex* positive group. *Demodex* infestation is possibly one of the key contributors to the pathogenesis of MGD related dry eye patients. In addition, it was associated with a higher prevalence of chalazion in those patients.

## Data Availability Statement

The original contributions presented in the study are included in the article/supplementary material, further inquiries can be directed to the corresponding author.

## Ethics Statement

The studies involving human participants were reviewed and approved by Beijing Tongren Hospital, Capital Medical University, Beijing, China. The patients/participants provided their written informed consent to participate in this study.

## Author Contributions

YH: analysis and interpretation of data, drafting the article, final approval of the version to be published, and agreement to be accountable for all aspects of the work. XZ and JB: acquisition of data, drafting the article, final approval of the version to be published, and agreement to be accountable for all aspects of the work. LT: analysis and interpretation of data, revising it critically for important intellectual content, final approval of the version to be published, and agreement to be accountable for all aspects of the work. YJ: substantial contributions to conception and design, revising it critically for important intellectual content, final approval of the version to be published, and agreement to be accountable for all aspects of the work. All authors contributed to the article and approved the submitted version.

## Funding

This research was supported by the Youth Beijing Scholars program, Natural Science Foundation of China (8217040526, 8217040515), and the Open Research Fund from Beijing Advanced Innovation Center for Big Data-Based Precision Medicine, Beijing Tongren Hospital, Beihang University and Capital Medical University (BHTR-KFJJ-202001).

## Conflict of Interest

The authors declare that the research was conducted in the absence of any commercial or financial relationships that could be construed as a potential conflict of interest.

## Publisher's Note

All claims expressed in this article are solely those of the authors and do not necessarily represent those of their affiliated organizations, or those of the publisher, the editors and the reviewers. Any product that may be evaluated in this article, or claim that may be made by its manufacturer, is not guaranteed or endorsed by the publisher.
